# Retro- and orthonasal olfactory function in relation to olfactory bulb volume in patients with hypogonadotrophic hypogonadism^☆^

**DOI:** 10.1016/j.bjorl.2017.07.009

**Published:** 2017-08-24

**Authors:** Murat Salihoglu, Onuralp Kurt, Seyid Ahmet Ay, Kamil Baskoy, Aytug Altundag, Muzaffer Saglam, Ferhat Deniz, Hakan Tekeli, Arif Yonem, Thomas Hummel

**Affiliations:** a GATA Haydarpasa Training Hospital, Department of Otorhinolaryngology, Istanbul, Turkey; b GATA Haydarpasa Training Hospital, Department of Endocrinology and Metabolism, Istanbul, Turkey; c Istanbul Surgical Hospital, Department of Otorhinolaryngology, Istanbul, Turkey; d GATA Haydarpasa Training Hospital, Department of Radiology, Istanbul, Turkey; e GATA Haydarpasa Training Hospital, Department of Neurology, Istanbul, Turkey; f University of Dresden Medical School, Smell & Taste Interdisciplina Center, Department of Otorhinolaryngology, Dresden, Germany

**Keywords:** Olfaction, Gustatory function, Hypogonadotropic hypogonadism, Olfactory bulb, Olfato, Função gustativa, Hipogonadismo hipogonadotrófico, Bulbo olfatório

## Abstract

**Introduction:**

Idiopathic hypogonadotrophic hypogonadism with an olfactory deficit is defined as Kallmann syndrome and is distinct from normosmic idiopathic hypogonadotrophic hypogonadism.

**Objective:**

Because olfactory perception not only consists of orthonasally gained impressions but also involves retronasal olfactory function, in this study we decided to comprehensively evaluate both retronasal and orthonasal olfaction in patients with idiopathic hypogonadotrophic hypogonadism.

**Methods:**

This case-control study included 31 controls and 45 idiopathic hypogonadotrophic hypogonadism patients. All participants whose olfactory and taste functions were evaluated with orthonasal olfaction (discrimination, identification and threshold), retronasal olfaction, taste function and olfactory bulb volume measurement. The patients were separated into three groups according to orthonasal olfaction: anosmic idiopathic hypogonadotrophic hypogonadism, hyposmic idiopathic hypogonadotrophic hypogonadism and normosmic idiopathic hypogonadotrophic hypogonadism.

**Results:**

Discrimination, identification and threshold scores of patients with Kallmann syndrome were significantly lower than controls. Threshold scores of patients with normosmic idiopathic hypogonadotrophic hypogonadism. were significantly lower than those of controls, but discrimination and identification scores were not significantly different. Retronasal olfaction was reduced only in the anosmic idiopathic hypogonadotrophic hypogonadism group compared to controls. Identification of bitter, sweet, sour, and salty tastes was not significantly different when compared between the anosmic, hyposmic, and normosmic idiopathic hypogonadotrophic hypogonadism groups and controls. Olfactory bulb volume was lower bilaterally in all patient groups when compared with controls. The olfactory bulb volume of both sides was found to be significantly correlated with threshold, discrimination and identification scores in idiopathic hypogonadotrophic hypogonadism patients.

**Conclusion:**

1) There were no significant differences in gustatory function between controls and idiopathic hypogonadotrophic hypogonadism patients; 2) retronasal olfaction was reduced only in anosmic patients but not in orthonasally hyposmic participants, possibly indicating presence of effective compensatory mechanisms; 3) olfactory bulb volumes were highly correlated with olfaction scores in the hypogonadotrophic hypogonadism group. The current results indicate a continuum from anosmia to normosmia in idiopathic hypogonadotrophic hypogonadism patients.

## Resumo

**Introdução:**

O hipogonadismo hipogonadotrófico idiopático com déficit olfatório é definido com síndrome de Kallmann e é distinto de hipogonadismo hipogonadotrófico idiopático normósmico.

**Objetivo:**

Pelo fato de a percepção olfativa não apenas consistir em impressões obtidas ortonasalmente, mas também envolver a função olfativa retronasal, neste estudo decidimos avaliar de maneira abrangente o olfato retronasal e ortonasal em pacientes com hipogonadismo hipogonadotrófico idiopático.

**Método:**

Este estudo caso-controle incluiu 31 controles e 45 pacientes com hipogonadismo hipogonadotrófico idiopático. Todos os participantes tiveram as funções olfativas e de paladar avaliadas com olfação ortonasal (discriminação, identificação e limiar), olfação retronasal, função do paladar e medida do volume do bulbo olfatório. Os pacientes foram separados em três grupos de acordo com a olfação ortonasal: hipogonadismo hipogonadotrófico idiopático anósmico, hipogonadismo hipogonadotrófico idiopático hipósmico e hipogonadismo hipogonadotrófico idiopático normósmico.

**Resultados:**

Os escores de discriminação, identificação e limiar de pacientes com síndrome de Kallmann foram significativamente menores do que os controles. Os escores dos limiares de pacientes com hipogonadismo hipogonadotrófico idiopático foram significativamente menores do que os dos controles, mas os escores de discriminação e identificação não foram significativamente diferentes. A olfação retronasal foi reduzida apenas no grupo hipogonadismo hipogonadotrófico idiopático anósmico em comparação com os controles. A identificação de gostos amargos, doces, azedos e salgados não foi significativamente diferente quando comparada entre os grupos e controles de hipogonadismo hipogonadotrófico idiopático anósmicos, hipósmicos e normósmicos. O volume do bulbo olfatório foi menor bilateralmente em todos os grupos de pacientes quando comparado com os controles. O volume do bulbo olfatório de ambos os lados foi significativamente correlacionado com os escores de limiar, discriminação,identificação em pacientes com hipogonadismo hipogonadotrófico idiopático.

**Conclusão:**

1) Não houve diferenças significativas na função gustativa entre controles e pacientes com hipogonadismo hipogonadotrófico idiopático; 2) A olfação retronasal foi reduzida apenas em pacientes anosmáticos, mas não em participantes ortonasalmente hipósmicos, possivelmente indicou presença de mecanismos compensatórios efetivos; 3) Os volumes do bulbo olfatório foram altamente correlacionados com os escores de olfação no grupo hipogonadismo hipogonadotrófico. Os resultados atuais indicam um contínuo da anosmia à normosmia em pacientes com hipogonadismo hipogonadotrófico idiopático.

## Introduction

Pulsatile secretion of gonadotropin releasing hormone (GnRH) from the hypothalamus causes release of the pituitary gonadotropins, luteinizing hormone (LH) and follicle stimulating hormone (FSH). Idiopathic hypogonadotrophic hypogonadism (IHH) refers to an evident hypogonadism phenotype with low levels of serum gonadotropins without anatomic problems in the hypothalamo-pituitary axis.^1^ Prevalence of IHH is estimated to be between 1/4000–1/10,000 in males and 1/40,000 in females.^2^

GnRH-secreting neurons and olfactory neurons share an embryologic migration pathway. These two neuron groups originate from the embryonic olfactory placode. Axons from olfactory receptor neurons and GnRH-secreting neurons migrate through the cribriform plate, where axons from olfactory receptor neurons synapse in the olfactory bulb (OB). Further migration of GnRH-secreting neurons targets the mediobasal hypothalamus, where functional pulsatile GnRH secretion occurs.^3^ Abnormal development of the olfactory placode leads to improper development of the OB and olfactory sulcus aplasia or hypoplasia of the OB and olfactory tracts is frequently associated with anosmia.

Hypogonadotrophic hypogonadism (HH) with an olfactory deficit is defined as Kallmann syndrome (KS) and is distinct from normosmic HH. However both diseases could share a common genetic etiology.^1^ Older studies have shown that deletion of the KAL1 gene is related to KS.^4^, ^5^ This deletion causes defects in migration of GnRH-secreting neurons in embryogenesis. Similarly, deletions in genes functioning on the fibroblast growth factor signaling pathway (FGF8 and FGFR1) may cause KS. KS may also result from deletions in genes functioning on the PROK2 signaling pathway (PROK2 and PROKR2) and CHD7 pathway. These gene deletions can also be seen in normosmic HH patients.^3^ Genes affecting secretion of GnRH (GNRHR, TAC3, KISS1, and KISS1R) have also been found in both hyposmic and normosmic HH patients. These studies indicate that normosmic, hyposmic, and anosmic HH have their basis in the same pathogenesis, but different expressions of related genes cause clinical variances.

Most previous studies on HH patients have used various psychophysiologic methods to assess olfactory function.^3^, ^6^, ^7^ Because olfactory perception not only consists of orthonasally gained impressions but also involves retronasal olfactory function, we decided to comprehensively evaluate both retronasal and orthonasal olfaction. Possible differences in gustatory function of HH patients were also investigated.

## Methods

The present investigation was designed as a case-control study and was approved by the Clinical Research Ethics Committee of Haydarpasa Training Hospital. All subjects gave written informed consent. Hypogonadotropic hypogonadism patients were recruited from our institution's endocrinology department and were selected consecutively for further evaluation. Serum free testosterone (ng/dL), serum total testosterone (ng/dL), serum sex hormone binding globulin (nmoL/L), FSH (mIU/mL), and LH (mIU/mL) were measured. Bilateral testis volumes (mL) were evaluated using scrotal ultrasonography. The study group consisted of men with the following characteristics: 18 years or older, clinical signs and symptoms of hypogonadism, serum testosterone levels under 100 ng/dL, low or normal levels of gonadotropins, normal serum levels of other anterior pituitary hormones, and absence of anatomic anomaly in the hypothalamic and pituitary areas confirmed with magnetic resonance imaging.

### Orthonasal olfactory testing

Psychophysical olfactory testing was conducted using the commercially available Sniffin’ Stick test kit, in which odors are presented via felt-tip pens.^8^, ^9^, ^10^ After opening the pen cap, each odorant was smelled by the patient for approximately 3 s. Olfactory tests were divided into three parts. The first part was the threshold test (T), during which increasing concentrations of phenyl-ethyl alcohol were presented. The second part was the odor discrimination test (D), consisting of 16 triplets with two different odors per triplet. The third part was an odor Identification test (I) using 16 common odors; participants were asked to choose one of four choices to identify each particular odor. Each test's maximum score was 16, and the maximum composite score was 48 (TDI score: total of threshold, discrimination, and identification scores). Normal values for the TDI composite score are > 30.5, with a cut-off between anosmia and hyposmia at 16.5.^11^ According to their TDI scores, study participants were diagnosed as functionally anosmic (hereafter called “anosmic”), hyposmic, or normosmic.

### Retronasal olfactory testing

For retronasal olfactory testing, a standardized, validated test was used.^12^ The test includes 20 items and is based on the identification of odorized powders or granules (e.g., ground instant coffee, cinnamon, or mushrooms) presented to the oral cavity. The substances were applied to the midline of the tongue with disposable plastic sticks. Participants were free to sample as much stimulant as necessary for identification. This approach minimized the problem of standardizing the area of stimulation and differences in sizes of participants’ tongues and oral cavities. In a typical trial, the experimenter placed approximately 0.05 g of the test substance on the middle of the tongue. After administration of each powder, participants rinsed their mouths with tap water. The procedure was self-timed. Each substance was identified by means of a forced-choice procedure in which the participant selected one out of four verbal items presented together with each odor. The test result was the sum score of the correctly identified stimuli (maximum score 20).

### Taste function testing

Four basic tastants with four concentrations were used for taste evaluation. Each tastant was adsorbed on filter paper strips at each concentration (sweet: 0.4, 0.2, 0.1, 0.05 g/mL sucrose; sour: 0.3, 0.165, 0.09, 0.05 g/mL citric acid; salty: 0.25, 0.1, 0.04, 0.016 g/mL sodium chloride; bitter: 0.006, 0.0024, 0.0009, 0.0004 g/mL quinine hydrochloride). These impregnated filter paper strips were placed on the anterior tongue. Before every administration the mouth was rinsed with distilled water. Taste qualities were applied in a randomized fashion but in increasing order of intensity. Each participant was tested with each taste at every concentration for a total of 16 trials. Patients were asked to identify the taste from a list containing “sweet,” “sour,” “salty,” and “bitter” (forced multiple choice). Correct answers were added up for a taste score “(maximum score 16).^13^

### Olfactory bulb measurement

Examinations were performed using a 1.5 T magnetic resonance imaging (MRI) system (Avanto; Siemens, Erlangen, Germany) with a 12 channel head coil. Sections were angulated perpendicularly to the anterior base of the skull or cribriform plate. We used three-dimensional T2 sampling perfection with application of optimized contrast using different flip angle evolutions (SPACE) covering the anterior and middle segments of the base of the skull. Parameters of the three-dimensional T2 SPACE sequence were as follows: echo time, 224 ms; repetition time, 1440 ms; bandwidth, 349 Hz per pixel; field of view, 190 × 190; matrix, 520 × 512; slice thickness, 1 mm; no interslice gap.

Syngo MMWP software (Siemens Medical Solutions, Forchheim, Germany) was used to determine the volume of the right and left OBs. Volume measurements were performed by an experienced radiologist blinded to the clinical diagnosis. Before volumetric measurements, a midsagittal image of the OBs was chosen and the length of both OBs was measured. Afterward, measurements of the right and left olfactory bulb volumes (OBV) were performed through manual segmentation of the coronal slices by planimetric manual contouring (surface in pixels), then all pixels were added and multiplied by the *x*, *y*, and *z* axes (0.36 × 0.37 × 1 number of pixels) to obtain a volume in cubic millimeters.^14^

### Statistical analysis

Data analysis was performed using SPSS version 21.0 (SPSS Inc., Chicago, IL, USA). The normal distribution of considered variables was first evaluated using the Shapiro–Wilk test. Data are presented as means ± standard deviations for continuous variables and as the number of cases for categorical variables. In comparing two independent groups, the Student's *t*-test was used if the continuous variables were normally distributed, and the Mann–Whitney *U* test was used for non-normally distributed continuous variables. Categorical variables (proportions) were compared using the Chi-Square test. Patients were divided into three groups according to their Sniffin’ Stick scores (normosmia, hyposmia, and anosmia). In addition, to explore retronasal olfactory sensitivity in relation to group, age, gender, cigarette smoking, and alcohol consumption, data were submitted to analysis of variance using the general linear model with Bonferroni post hoc comparisons. Correlational analyses were calculated according to Pearson's correlation coefficient. The level of significance was set at *p* < 0.05.

## Results

Our study included 31 controls and 45 IHH patients. The groups did not differ significantly in terms of age, sex, or education level. As expected, the IHH patients had lower levels of serum free testosterone, total serum testosterone, FSH, LH, and lower right and left testis volumes (Table 1).

**Table 1:** Demographic, clinical and laboratory characteristics of patients and controls.^a^

	Controls (*n* = 31)	IHH (*n* = 45)	*p*-value
Age (years)	24.19 ± 3.89	23.64 ± 3.46	0.581
Education (years)	10.03 ± 2.80	9.84 ± 2.70	0.48
Serum free testosterone (ng/dL)	3.54 ± 11.17	1.79 ± 6.26	**<0.001**
Serum total testosterone (ng/dL)	113.33 ± 310.06	69.08 ± 176.15	**<0.001**
SHBG (nmoL/L)	27.93 ± 10.82	42.82 ± 27.23	**0.018**
FSH (mIU/mL)	3.53 ± 1.56	0.84 ± 1.24	**<0.001**
LH (mIU/mL)	4.28 ± 2.01	2.41 ± 13.82	**<0.001**
Right, testis volume (mL)	16.02 ± 1.49	2.07 ± 1.63	**<0.001**
Left, testis volume (mL)	16.11 ± 1.32	1.99 ± 1.44	**<0.001**

IHH, idiopathic hypogonadotropic hypogonadism; SHBG, sex hormone binding globulin; FSH, follicle stimulating hormone; LH, luteinizing hormone; *p*-value, controls vs. IHH. The values marked in bold are statistically significant (*p* < 0.05).

a Values are the mean and SD (standard deviations).

The patients were separated into three groups according to their TDI scores: anosmic IHH group (*n* = 14), hyposmic IHH group (*n* = 12), and normosmic IHH group (nIHH, *n* = 19). Olfactory (orthonasal and retronasal) and taste test results for patients and controls are shown in Table 2. Discrimination, identification, and threshold scores of patients with KS were significantly lower than those of controls. Threshold scores of patients with nIHH were significantly lower than those of controls, but discrimination and identification scores were not significantly different (odor threshold: 7.82 ± 0.42 vs. 8.69 ± 1.21, *p* = 0.01; TDI score: 34.24 ± 1.41 vs. 35.66 ± 2.09, *p* = 0.01). Retronasal olfaction was reduced only in the anosmic IHH group compared to controls (8.50 ± 3.01 vs. 16.84 ± 2.18, *p* = 0.001). The orthonasally hyposmic and nIHH groups were similar in retronasal olfaction when compared with controls. Identification of bitter, sweet, sour, and salty tastes was not significantly different when compared between the anosmic, hyposmic, and normosmic IHH groups and controls.

**Table 2:** Olfactory and taste test results of patients and controls.

Test (mean±SD)	Anosmia (*n* = 14) (KS)	Hyposmia (*n* = 12) (KS)	Normosmia (*n* = 19) (nIHH)	Controls (*n* = 31)	*P*_1_	*P*_2_	*P*_3_	*P*_4_	*P*_5_	*P*_6_
Threshold	2.57 ± 0.72	6.54 ± 0.82	7.82 ± 0.42	8.69 ± 1.21	**<0.001**	**<0.001**	**0.01**	**<0.001**	**<0.001**	**<0.001**
Discrimination	3.71 ± 0.91	8 ± 0.74	12.89 ± 0.88	13.45 ± 1.06	**<0.001**	**<0.001**	0.07	**<0.001**	**<0.001**	**<0.001**
Identification	4.93 ± 1.14	9.42 ± 0.67	13.58 ± 0.84	13.52 ± 1	**<0.001**	**<0.001**	0.75	**<0.001**	**<0.001**	**<0.001**
TDI	11.21 ± 2.46	23.96 ± 1.62	34.24 ± 1.41	35.66 ± 2.09	**<0.001**	**<0.001**	**0.01**	**<0.001**	**<0.001**	**<0.001**
Bitter	3.36 ± 0.5	3.42 ± 0.51	3.42 ± 0.51	3.42 ± 0.5	0.70	0.99	0.99	0.76	0.72	0.98
Sweet	3.29 ± 0.61	3.25 ± 0.75	3.32 ± 0.67	3.29 ± 0.64	0.95	0.93	0.87	0.98	0.84	0.84
Salt	3.36 ± 0.63	3.33 ± 0.65	3.37 ± 0.68	3.35 ± 0.66	0.97	0.89	0.92	0.91	0.90	0.84
Sour	3.29 ± 0.61	3.25 ± 0.45	3.32 ± 0.58	3.32 ± 0.54	0.89	0.62	1.00	0.78	0.90	0.66
Total taste	13.29 ± 1.82	13.25 ± 1.86	13.42 ± 1.98	13.39 ± 2.01	0.94	0.86	0.99	0.94	0.93	0.80
Retronasal	8.50 ± 3.01	16.33 ± 1.72	16.47 ± 2.44	16.84 ± 2.18	**<0.001**	0.21	0.62	**<0.001**	**<0.001**	0.46

KS, Kallmann syndrome; nIHH, normosmic idiopathic hypogonadotropic hypogonadism; SD, standard deviations; *P*_1_ value, anosmia vs. controls; *P*_2_ value, hyposmia vs. controls; *P*_3_ value, normosmia vs. controls; *P*_4_ value, anosmia vs. hyposmia; *P*_5_ value, anosmia vs. normosmia; *P*_6_ value, hyposmia vs. normosmia. The values marked in bold are statistically significant (*p* < 0.05).

Olfactory bulbs were not seen in four patients in the anosmic group. OBV was lower bilaterally in all patient groups when compared with controls. However, the right OBV was not statistically different between the normosmic IHH group and the control group (Table 3).

**Table 3:** MRI Measurements^a^ of patients and controls.

OBV (mm^3^)	Anosmia (*n* = 14) (KS)	Hyposmia (*n* = 12) (KS)	Normosmia (*n* = 19) (nIHH)	Controls (*n* = 31)	*P*_1_	*P*_2_	*P*_3_	*P*_4_	*P*_5_	*P*_6_
Right	10.79 ± 10.09	38.88 ± 13.43	56.64 ± 8.91	65.52 ± 16.75	**<0.001**	**<0.001**	0.25	**<0.001**	**<0.001**	**<0.001**
Left	10.43 ± 10.13	39.12 ± 13.98	56.07 ± 9.15	66.61 ± 16.06	**<0.001**	**<0.001**	**0.03**	**<0.001**	**<0.001**	**<0.001**

MRI, magnetic resonance imaging; KS, Kallmann syndrome; nIHH, normosmic idiopathic hypogonadotropic hypogonadism; SD, standard deviations; *P*_1_ value, anosmia vs. controls; *P*_2_ value, hyposmia vs. controls; *P*_3_ value, normosmia vs. controls; *P*_4_ value, anosmia vs. hyposmia; *P*_5_ value, anosmia vs. normosmia; *P*_6_ value, hyposmia vs. normosmia. The values marked in bold are statistically significant (*p* < 0.05).

a Values are the mean and SD (standard deviations).

**Figure 1:**
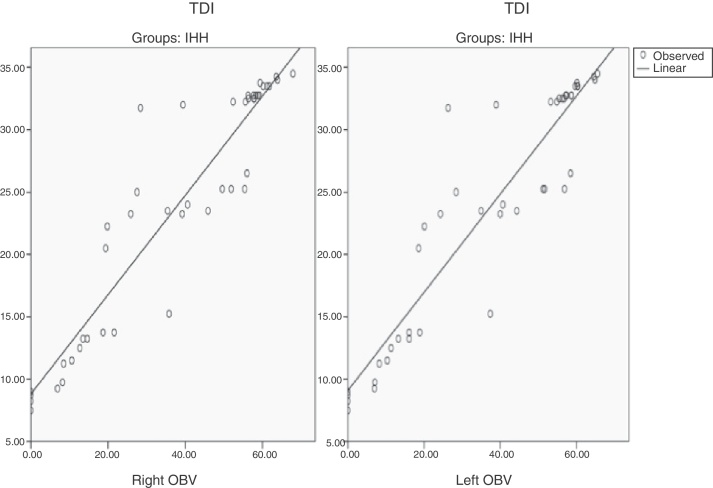
The correlation between OBV (olfactory bulb volume) and TDI (T, threshold; D, discrimination; I, identification) scores in IHH (idiopathic hypogonadotropic hypogonadism) patients.

**Figure 2:**
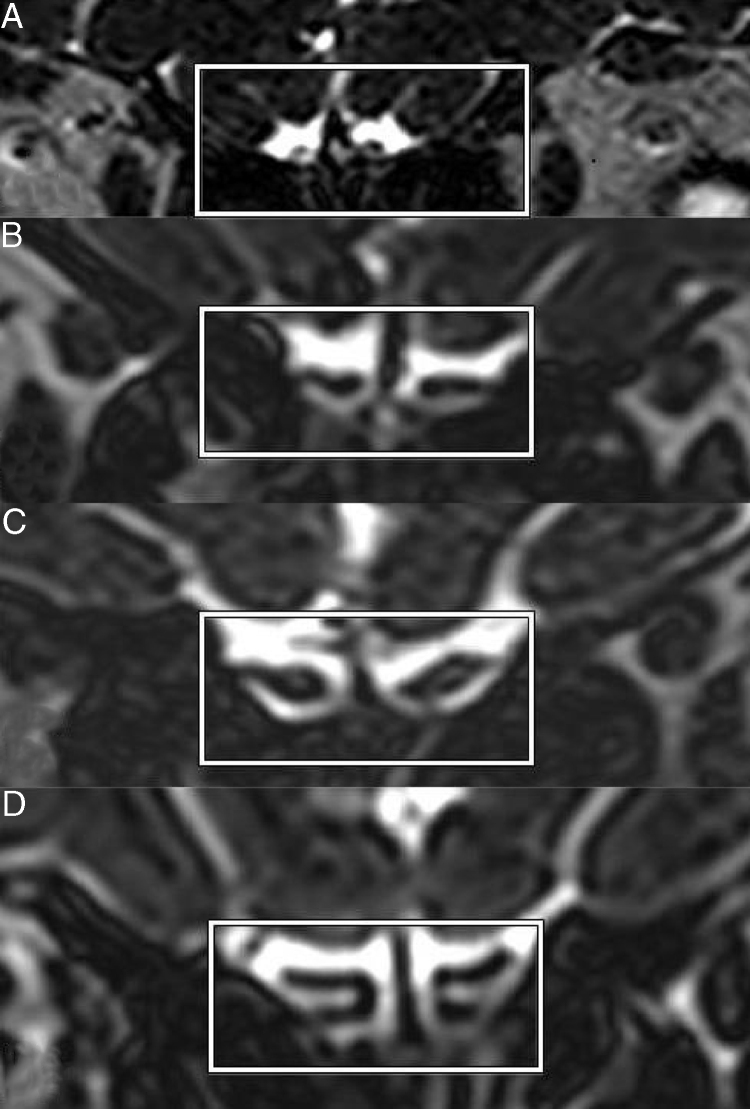
Coronal three-dimension T2 STIR (short tau inversion recovery) SPACE (sampling perfection with application-optimized contrasts using different flip angle evolution) images show olfactory bulbs of anosmia (A), hyposmia (B), normosmia (C), and control (D).

The OBV of both sides was found to be significantly correlated with TDI scores in IHH patients (right OBV-TDI, *r*: 0.934, *p* < 0.001; left OBV-TDI, *r*: 0.928, *p* < 0.001) (Table 4) (Figure 1, Figure 2). Serum indicators of hypogonadism, such as FSH, LH, serum free testosterone, serum total testosterone, and TDI correlation, were investigated; no significant correlations were found.

**Table 4:** Correlations between OBV and TDI scores in IHH.

Variable	IHH group (*n* = 45)	
	*r*^a^	*p*^a^
Right OBV-TDI	0.934	**<0.001**
Left OBV-TDI	0.928	**<0.001**

OBV, olfactory bulb volume; T, threshold; D, discrimination; I, identification; IHH, idiopathic hypogonadotropic hypogonadism. The values marked in bold are statistically significant (*p* < 0.05).

a Pearson correlation analysis.

## Discussion

Olfaction has been studied many times in HH patients. A difference in the sense of olfaction is the key finding to distinguish IHH from KS.^15^ Only a limited number of studies investigating taste in IHH could be found in the literature.^6^, ^16^ This study is one of the first studies evaluating retronasal and orthonasal olfaction and taste assessment together in HH patients. Categorization of patients as anosmic, hyposmic, or normosmic was made according to TDI scores for the Sniffin’ Stick test.^11^ Each group was compared with controls regarding odor threshold, odor discrimination, and identification of odors. Normosmics had lower threshold and TDI scores. Significantly lower olfactory scores even in normosmic HH patients compared to normosmic controls could be due to gradual expression of related HH genes.

Identification of bitter, salty, sweet, and sour tastes was tested between the control and study groups. Taste function was not significantly different between controls and any of the three HH subgroups. Ros et al. investigated smell and taste in Turner Syndrome patients; they found olfactory impairment in the study group but no significant difference for taste functions.^17^ The authors explained the olfactory impairment with genetic reasons and claimed that hormonal changes had no effect on gustatory function. That is possibly is the reason why taste function was very similar in the groups studied.

Flavor is a complex function, closely related to taste and to olfactory integrity, which ultimately enables food recognition, and enjoyment of food.^6^, ^18^ Retronasal olfaction was investigated in our study and was found to be significantly lower in the anosmic group in comparison to controls, but no significant difference was found between the hyposmic group and controls. Landis et al. compared orthonasal and retronasal olfaction and found that a significant proportion of orthonasally anosmic patients had normal function of retronasal olfaction.^19^ Author explained the difference with different vulnerability levels of anterior and posterior olfactory epithelium to damage or different procession of orthonasal and retronasal evocation of olfaction. Orthonasal and retronasal olfactory information has been shown to be differently processed on a cerebral level.^20^ Because the retronasal test also included gustatory stimulation (e.g., sweet vanilla sugar, salty celery salt, and bitter coffee powder),^19^, ^20^ our results may indicate that hyposmic patients were able to utilize their gustatory system to such an extent that their scores were comparable to controls. This may indicate that hyposmic HH patients develop some compensatory mechanisms to make up for their decreased olfactory sensitivity.

Patients experiencing olfactory loss complain mainly of flavor distortion and inability to identify food. However, KS patients generally do not complain of flavor dysfunctions. It is claimed that congenital anosmics might learn to concentrate on other aspects of foods to compensate for the lack of olfaction.^6^ Hasan et al. compared four KS patients to four controls using psychophysical smell tests, electrogustometry, and tests for flavor perception.^16^ They found lower smell scores with normal taste in KS patients, as expected. However, flavor perception is not typically a complaint in KS patients. Valle et al. studied 36 patients with IHH and found that 41.6% were hyposmic or anosmic.^1^ Hypoplasia or aplasia of the olfactory bulbs was found in 75% of the hyposmic/anosmic group but in only 7.6% of the normosmic group. Independent of olfactory status, midline defects and neurosensorial hearing loss occur at a high frequency in IHH patients, supporting the idea that KS and normosmic HH are in different positions on the spectrum of the same developmental disease.

Several studies already looked at possible prediction of olfactory function based on OBV.^21^ We measured OBV on both sides and searched for a relation between OBV and olfactory function. Olfactory bulb volumes were significantly smaller in all three study groups compared with controls, and we found good agreement between OBV and TDI scores in IHH patients. The exact reason for the decreased OBV in nIHH is not known. Jagtap et al. found a high prevalence of MRI abnormalities in nIHH patients, along with increased frequency of OBV hypoplasia, bilateral cerebellar atrophy, cleft lip, and cleft palate.^22^ They postulated that these patients may have subtle neuroanatomical olfactory abnormalities that may not compromise olfactory apparatus function (at least not at a younger age). Genetic studies of these patients would yield valuable information and may provide some clues regarding the reasons for the lower OBV in nIHH. Anik et al. studied olfactory bulb volumes in six KS patients and claimed that MRI imaging of the olfactory bulbs was strongly correlated with olfactometry results.^23^ Hudson et al. showed that the olfactory function scores of apparently normosmic subjects lay within the lower end of the normal ranges for control subjects, arguing for a subtle olfactory abnormality within this subset.^24^ Likewise, Vogl et al. demonstrated that a small percentage of apparently nIHH subjects had abnormal olfactory bulbs, and their smell tests were in the lower range of the normal distribution.^25^

## Conclusion

The current study provided the following major results: 1) there were no significant differences in gustatory function between controls and IHH patients; 2) retronasal olfaction was reduced only in anosmic patients but not in orthonasally hyposmic participants, possibly indicating presence of effective compensatory mechanisms; and 3) olfactory bulb volumes were highly correlated with olfaction scores in the HH group. The current results indicate a continuum from anosmia to normosmia in IHH patients.

## Conflicts of interest

The authors declare no conflicts of interest.

## References

[1] Della Valle E., Vezzani S., Rochira V., Granata A.R., Madeo B., Genovese E. (2013). Prevalence of olfactory and other developmental anomalies in patients with central hypogonadotropic hypogonadism. Front Endocrinol (Lausanne).

[2] Grumbach M.M. (2005). A window of opportunity: the diagnosis of gonadotropin deficiency in the male infant. J Clin Endocrinol Metab.

[3] Lewkowitz-Shpuntoff H.M., Hughes V.A., Plummer L., Au M.G., Doty R.L., Seminara S.B. (2012). Olfactory phenotypic spectrum in idiopathic hypogonadotropic hypogonadism: pathophysiological and genetic implications. J Clin Endocrinol Metab.

[4] Franco B., Guioli S., Pragliola A., Incerti B., Bardoni B., Tonlorenzi R. (1991). A gene deleted in Kallmann's syndrome shares homology with neural cell adhesion and axonal path-finding molecules. Nature.

[5] Legouis R., Hardelin J.P., Levilliers J., Claverie J.M., Compain S., Wunderle V. (1991). The candidate gene for the X-linked Kallmann syndrome encodes a protein related to adhesion molecules. Cell.

[6] Maione L., Cantone E., Nettore I.C., Cerbone G., De Brasi D., Maione N. (2016). Flavor perception test: evaluation in patients with Kallmann syndrome. Endocrine.

[7] Shin S.J., Sul Y., Kim J.H., Cho J.H., Kim G.H., Kim J.H. (2015). Clinical, endocrinological, and molecular characterization of Kallmann syndrome and normosmic idiopathic hypogonadotropic hypogonadism: a single center experience. Ann Pediatr Endocrinol Metab.

[8] Tekeli H., Altundağ A., Salihoğlu M., Cayönü M., Kendirli M.T. (2013). The applicability of the “Sniffin’ Sticks” olfactory test in a Turkish population. Med Sci Monit.

[9] Deniz F., Ay S.A., Salihoglu M., Kurt O., Baskoy K., Altundag A. (2016). Thyroid hormone replacement therapy improves olfaction and taste sensitivity in primary hypothyroid patients: a prospective randomised clinical trial. Exp Clin Endocrinol Diabetes.

[10] Baskoy K., Ay S.A., Altundag A., Kurt O., Salihoglu M., Deniz F. (2016). Is There any effect on smell and taste functions with levothyroxine treatment in subclinical hypothyroidism?. PLOS ONE.

[11] Hummel T., Kobal G., Gudziol H., Mackay-Sim A. (2007). Normative data for the “Sniffin’ Sticks” including tests of odor identification, odor discrimination, and olfactory thresholds: an upgrade based on a group of more than 3,000 subjects. Eur Arch Otorhinolaryngol.

[12] Salihoglu M., Altundag A., Cayonu M., Tekeli H. (2014). An investigation of retronasal testing of olfactory function in a Turkish population. Med Sci Monit.

[13] Mueller C., Kallert S., Renner B., Stiassny K., Temmel A.F., Hummel T. (2003). Quantitative assessment of gustatory function in a clinical context using impregnated “taste strips”. Rhinology.

[14] Saglam M., Salihoglu M., Tekeli H., Altundag A., Sivrioglu A.K., Cayonu M. (2014). Is there an association between olfactory bulb volume and the Keros type of fossa olfactoria?. J Craniofac Surg.

[15] Forni P.E., Wray S. (2015). GnRH, anosmia and hypogonadotropic hypogonadism – where are we?. Front Neuroendocrinol.

[16] Hasan K.S., Reddy S.S., Barsony N. (2007). Taste perception in kallmann syndrome, a model of congenital anosmia. Endocr Pract.

[17] Ros C., Alobid I., Centellas S., Balasch J., Mullol J., Castelo-Branco C. (2012). Loss of smell but not taste in adult women with Turner's syndrome and other congenital hypogonadisms. Maturitas.

[18] Schiffman S.S. (1997). Taste and smell losses in normal aging and disease. JAMA.

[19] Landis B.N., Welge-Luessen A., Brämerson A., Bende M., Mueller C.A., Nordis S. (2009). Taste Strips” – a rapid, lateralized, gustatory bedside identification test based on impregnated filter papers. J Neurol.

[20] Small D.M., Gerber J.C., Mak Y.E., Hummel T. (2005). Differential neural responses evoked by orthonasal versus retronasal odorant perception in humans. Neuron.

[21] Rombaux P., Duprez T., Hummel T. (2009). Olfactory bulb volume in the clinical assessment of olfactory dysfunction. Rhinology.

[22] Jagtap V.S., Sarathi V., Lila A.R., Nair S., Bukan A., Sankhe S.S. (2013). An objective olfactory evaluation and its correlation with magnetic resonance imaging findings in Asian Indian patients with idiopathic hypogonadotropic hypogonadism. Endocr Pract.

[23] Anık A., Çatlı G., Abacı A., Güleryüz H., Güdücü Ç., Öniz A. (2015). Olfactory dysfunction in children with Kallmann syndrome: relation of smell tests with brain magnetic resonance imaging. Hormones (Athens).

[24] Hudson R., Laska M., Berger T., Heye B., Schopohl J., Danek A. (1994). Olfactory function in patients with hypogonadotropic hypogonadism: an all-or-none phenomenon?. Chem Senses.

[25] Vogl T.J., Stemmler J., Heye B., Schopohl J., Danek A., Bergman C. (1994). Kallman syndrome versus idiopathic hypogonadotropic hypogonadism at MR imaging. Radiology.

